# Reduced Fluorescent Protein Switching Fatigue by Binding-Induced Emissive State Stabilization

**DOI:** 10.3390/ijms18092015

**Published:** 2017-09-20

**Authors:** Thijs Roebroek, Sam Duwé, Wim Vandenberg, Peter Dedecker

**Affiliations:** Laboratory for Nanobiology, Department of Chemistry, KU Leuven, Celestijnenlaan 200G, 3001 Leuven, Belgium; thijs.roebroek@kuleuven.be (T.R.); sam.duwe@kuleuven.be (S.D.); wim.vandenberg@kuleuven.be (W.V.)

**Keywords:** fluorescent proteins, reversible photoswitching, photochromism, switching fatigue, contrast, nanobody, rsGreen, spectroscopy

## Abstract

Reversibly switchable fluorescent proteins (RSFPs) enable advanced fluorescence imaging, though the performance of this imaging crucially depends on the properties of the labels. We report on the use of an existing small binding peptide, named Enhancer, to modulate the spectroscopic properties of the recently developed rsGreen series of RSFPs. Fusion constructs of Enhancer with rsGreen1 and rsGreenF revealed an increased molecular brightness and pH stability, although expression in living *E. coli* or HeLa cells resulted in a decrease of the overall emission. Surprisingly, Enhancer binding also increased off-switching speed and resistance to switching fatigue. Further investigation suggested that the RSFPs can interconvert between fast- and slow-switching emissive states, with the overall protein population gradually converting to the slow-switching state through irradiation. The Enhancer modulates the spectroscopic properties of both states, but also preferentially stabilizes the fast-switching state, supporting the increased fatigue resistance. This work demonstrates how the photo-physical properties of RSFPs can be influenced by their binding to other small proteins, which opens up new horizons for applications that may require such modulation. Furthermore, we provide new insights into the photoswitching kinetics that should be of general consideration when developing new RSFPs with improved or different photochromic properties.

## 1. Introduction

During the past decades, fluorescent proteins (FPs) have been widely used as tools in fluorescence microscopy to address a broad array of biological and biomedical questions. At the heart of this success is the non-invasive and sensitive nature of fluorescence microscopy, combined with the outstanding selectivity and labeling properties of the fluorophores [1]. The widespread success of fluorescent proteins in imaging has led to an impressive toolbox of FPs that covers a gamut of possible colors and encompasses a large range of photo-physical properties [2,3].

The discovery and development of photoresponsive FPs has been of particular interest [1,4]. This class of “smart” fluorescent proteins possesses controllable dynamic fluorescence emission, which enables their use for several sub-diffraction imaging techniques such as (fluorescence) photoactivated localization microscopy ((f)PALM) [5,6], reversible saturable optical linear fluorescence transitions (RESOLFT) [7,8,9], non-linear structured illumination microscopy (NL-SIM) [10,11], and photochromic stochastic optical fluctuation imaging (pcSOFI) [12,13]. These sub-diffraction techniques rely heavily on the performance of the fluorophores [14]. Consequently, plenty of effort has been devoted to creating more optimized photoresponsive FPs, as well as to achieving a fundamental understanding of the mechanisms through which this “smart” behavior is defined [15,16,17,18,19,20,21].

A recent example is the development of the rsGreen series of reversibly switchable fluorescent proteins (RSFPs), based on the enhanced green fluorescent protein (EGFP) [22]. The development of these and many other fluorescent proteins has established that the immediate chromophore microenvironment crucially affects the spectroscopy and photochemistry of these labels [2,22]. Intriguingly, this dependence on the protein structure is not just limited to the residues closest to the chromophore, as different research groups have shown that the spectroscopic properties of FPs can be manipulated through interactions with other molecules, often proteins, further removed from the chromophore environment. The postulated mechanism for these modulators often relies on an influence on protein dynamics and rearrangements which indirectly change the chromophore environment, thereby affecting the photo-physical properties [23,24,25,26,27,28]. Nanobodies, for example, tightly bind GFP-like fluorescent proteins, and have been shown to modulate the spectral properties and pH sensitivity of these labels in doing so. One of these, the so-called “Enhancer” nanobody, is of particular interest as it results in increased fluorescence and improved pH stability of wild type (wt)GFP, superfolder (sf)GFP and EGFP upon binding [23,29]. In addition, the nanobody could be fused directly to the fluorescent protein itself, leading to a single label with improved spectroscopic properties that can directly benefit live cell imaging [29].

We hypothesized that such effects could similarly lead to altered properties in RSFPs that may improve their performance in advanced fluorescence imaging. In this work we therefore set out to examine the effect of the Enhancer nanobody on RSFPs from the rsGreen series.

## 2. Results

### 2.1. Effect of Enhancer on rsGreen Brightness

**Figure 1:**
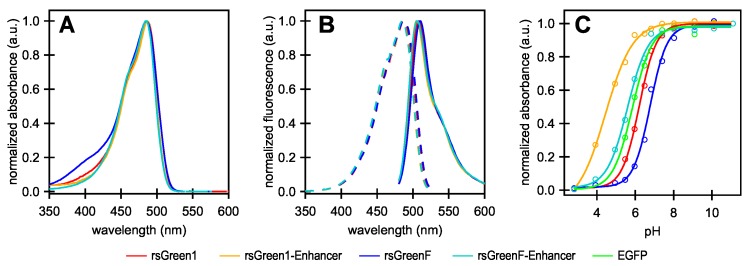
Absorption, excitation and emission spectra of rsGreens and Enhancer constructs. (**A**) Normalized absorbance spectra; (**B**) excitation (dashes) and emission (solid) spectra; (**C**) pH titration curves showing the absorption maximum at different pH values of the indicated labels. All graphs were normalized to their maximum absorption, excitation or emission. EGFP: enhanced green fluorescent protein; a.u.: arbitrary units.

Following the strategy outlined in the work by Eshaghi et al. (2015), we started by creating two chimeric proteins in which rsGreen1 and rsGreenF were directly fused to the N-terminus of the Enhancer nanobody. We expressed and purified the resulting constructs and performed an in vitro spectroscopic characterization (Figure 1 and Table 1). All of the examined proteins display absorption and excitation maxima at 485–488 nm and emission maxima at 505–510 nm, consistent with other green fluorescent proteins, with the overall excitation and emission spectra displaying little change upon fusion to the Enhancer nanobody. As was observed for previous Enhancer fusions, an increase in molecular brightness was found (23% for rsGreen1 and 21% for rsGreenF). The quantum yields of fluorescence appeared to be largely unchanged and the brightness enhancement largely results from an increase in absorption. This increase is mainly due to the reduced pH sensitivity (lower $pK_{a}$) of the nanobody fusions, resulting in a higher occupancy of the anionic absorbing form and increased apparent extinction coefficients observed at physiological pH. However, when we extrapolate these extinction coefficients to those of the fully deprotonated form, a net increase in extinction coefficient still remains (13% for rsGreen1 and 11% for rsGreenF). This observation indicates the presence of additional effects of the Enhancer nanobody on the FP’s brightness in addition to those stemming from $pK_{a}$ differences.

**Figure 2:**
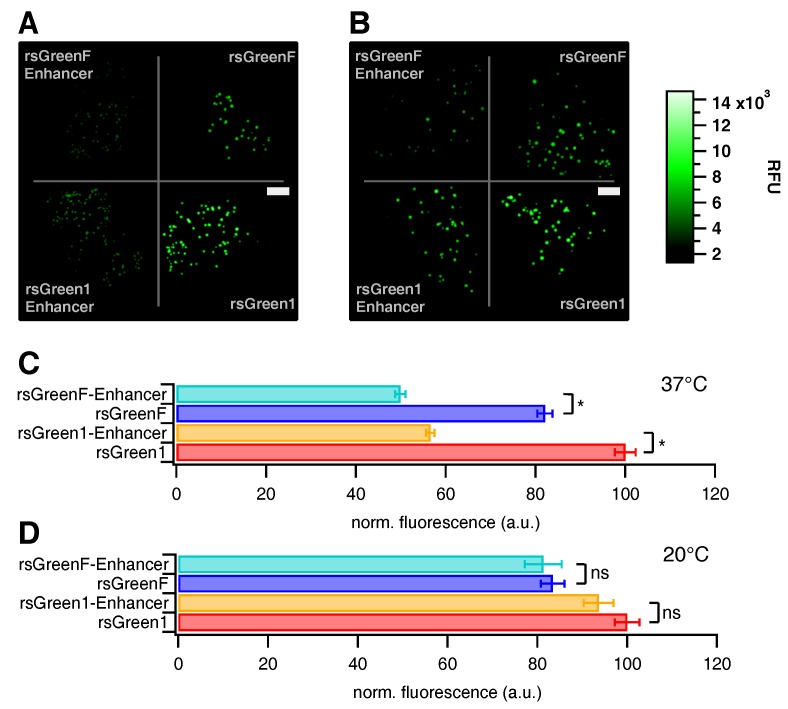
Effect of Enhancer nanobody fusion on the brightness of *E. coli* colonies expressing the different labels. (**A**,**B**) Fluorescence images of *E. coli* colonies expressing indicated labels, grown at 37 ${}^{\circ }$C (**A**); and 20 ${}^{\circ }$C (**B**) (RFU = relative fluorescence units, scale bar (white rectangles) = 1 cm). (**C**,**D**) Average colony fluorescence scaled to 100 for rsGreen1 for 37 ${}^{\circ }$C (**C**); and 20 ${}^{\circ }$C (**D**) plates. Error bars represent standard error on the mean. *: significant differences (p≤0.01). ns: not significant.

Following the in vitro characterization we assessed the influence of the Enhancer nanobody in situ. *E. coli* colonies expressing the different FPs were prepared on growth plates, which were visualized on a home-built colony imaging system. There was a notable decrease in total colony fluorescence upon fusion of the rsGreens with the Enhancer nanobody when grown one day at 37 ${}^{\circ }$C (Figure 2A,C), though no change was apparent when grown three days at 20 ${}^{\circ }$C (Figure 2B,D). This demonstrates an influence of Enhancer fusion on the folding and maturation of the FPs. The average colony fluorescence was found to be 43% lower for rsGreen1 and 39% lower for rsGreenF (Table 1 and Table S1). Similarly, we witnessed an average decrease in fluorescence for transfected HeLa cells (31% and 36% respectively). Compared to the measured spectroscopic properties, these data imply that improvements found upon Enhancer-binding in vitro do not necessarily translate directly to better live-cell fluorescence properties.

### 2.2. Effect of Enhancer on rsGreen Photoswitching

**Figure 3:**
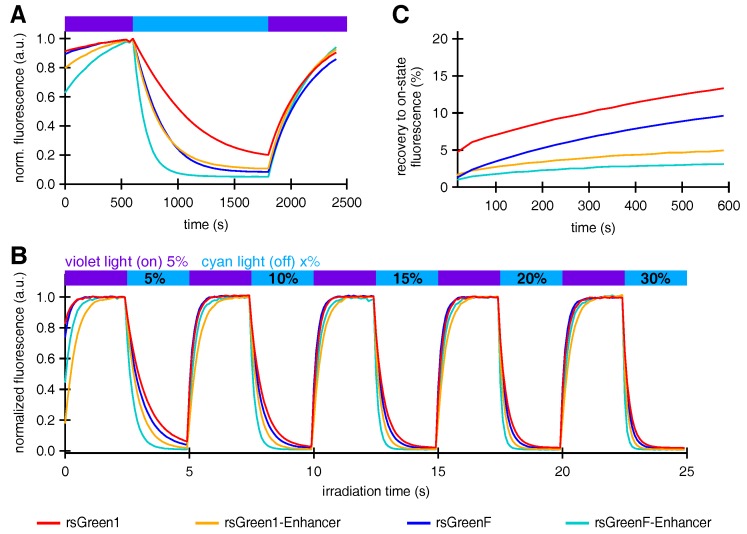
The effect of Enhancer nanobody binding on the photoswitching behavior of rsGreens. (**A**) Normalized average fluorescence of *E. coli* colonies expressing indicated FPs upon irradiation with violet, blue and violet light. (**B**) Normalized average fluorescence in HeLa cells upon irradiation with violet and cyan light with indicated preset powers. (**C**) Spontaneous, thermal recovery of the fluorescence in HeLa cells after off-switching with cyan light. a.u.: arbitrary units.

We proceeded to study the effect of Enhancer nanobody fusion on the reversible photoswitching behavior of the rsGreens. *E. coli* colonies expressing the rsGreens and Enhancer fusions all displayed negative photoswitching with faster off-switching for rsGreenF compared to rsGreen1 (Figure 3A), as expected from previous observations [22]. Remarkably, the presence of the Enhancer nanobody increased the off-switching rate for both rsGreen1 and rsGreenF. A similar trend was observed when the constructs were expressed in HeLa cells, also revealing the lower light intensities and irradiation times required by the Enhancer fusions to achieve complete off-switching (Figure 3B). A detailed analysis of this effect revealed that about half of the increase in off-switching speed could be attributed to the increase in absorption and reduction in pKa, while the remainder reflects an intrinsic change in the protein propensity to switching (see Appendix A and Table A1). Additionally, the spontaneous recovery from the off- to the on-state was greatly diminished when the Enhancer nanobody was fused to the rsGreens (Figure 3C). Taken together, these observations imply a large effect of the bound nanobody on the structure and function of the photochromic FPs. This is consistent with previous literature that reported on an intricate link between protein-protein association and the photoswitching process in RSFPs [31].

### 2.3. Effect of Enhancer on rsGreen Throughout Multiple Switching Cycles

**Figure 4:**
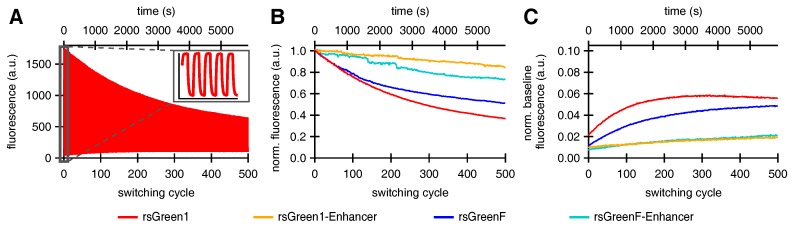
The effect of Enhancer nanobody binding on the repeated photoswitching behavior of rsGreens in HeLa cells (500 cycles): (**A**) representative fluorescence photoswitching trace of rsGreen1, inset shows the initial five switching cycles; (**B**) signal of the maximally on-switched frame for each switching cycle scaled to the initial on-state fluorescence; and (**C**) baseline fluorescence (maximally off-switched frame) as fraction of the initial on-state fluorescence.

In accordance with previous reports we expected that faster off-switching would result in a lower switching fatigue (higher number of achievable switching cycles) and a high contrast between the fluorescent and non-fluorescent states [22,32]. To test this hypothesis, we analyzed the repeated photoswitching behavior of the free RSFPs and the Enhancer fusions cytosolically expressed in HeLa cells (Figure 4A and Figure S1). When we examined the level of the on-state fluorescence over the course of 500 switching cycles, it became apparent that the Enhancer fusions displayed a reduced loss in fluorescence intensity, as expected (Figure 4B). Additionally, the data revealed that baseline fluorescence levels increased during the experiment. This effect was less prominent for the Enhancer fusions, leading to an improved on/off contrast (Figure 4C). A similar rise in baseline fluorescence was previously reported for GMars-Q, rsEGFP2 and rsEGFP(N205S) and claimed to be the result of RSFPs getting trapped in a non-switching fluorescent state [33].

**Figure 5:**
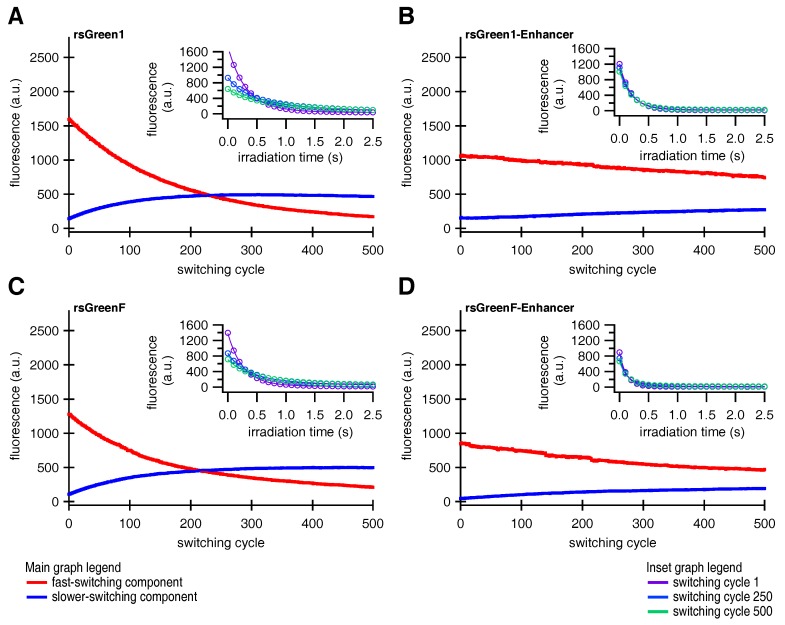
Global fits describing a two-species model for photoswitching in HeLa cells. Contribution of the two switching species of (**A**) rsGreen1, (**B**) rsGreen1-Enhancer, (**C**) rsGreenF, (**D**) rsGreenF-Enhancer and their respective fits (Appendix B). Red curves represent the fast-switching species A, blue curves the slower-switching species B. Inset: single off-switching decays and their respective fits for switching cycles 1 (purple), 250 (light blue) and 500 (green).

We made two more intriguing observations: we found that the fluorescence time trace of a single switching cycle required at least a bi-exponential function for adequate fitting, and the overall fluorescence switching became slower after repeated switching cycles (insets in Figure 5). Previous reports on other RSFPs already described the existence of two populations with different switching kinetics [31]. Based on these observations, we hypothesized that the fluorescent proteins could exist in two different emissive states, A and B, that each display distinct switching kinetics. We therefore designed a model function for the off-switching (discussed in Appendix B.1), that was applied to globally fit the 500 individual off-switching fluorescence decays for each of the rsGreens and their Enhancer-fused analogues. The parameters describing the photoswitching behavior of both species were kept constant for all 500 cycles. Our model fitted the data well, revealing a varying contribution of both states throughout the experiment that additionally suggested light-driven interconversion between these states (Figure 5). The fast-switching species A is initially dominant but is progressively depopulated, while the slower-switching species B initially grows in abundance before leveling off and disappearing as well, presumably due to photodestruction. This behavior readily explains the increasing baseline fluorescence witnessed throughout the photoswitching experiments as being due to accumulation of the slower-switching species B, which does not feature complete switching to a non-fluorescent state ($F_{B}$, Table A2), without the need to involve a separate non-switching form [33]. In a next step, we modeled the evolution of both states by assuming interconversion throughout the experiment combined with photodestruction occurring in both states (discussed in Appendix B.2). Our model matched well with the observed varying presence of both species, as can be seen from the fits in Figure 5.

Looking at the data for the two-state model, the main effect of the nanobody is to slow down the decrease in the population of species A, suggesting that it stabilizes the formation of this state. With species A being the predominant state, such stabilization could rationalize the increased resistance to photoswitching fatigue that was observed upon Enhancer binding. In addition, previous literature suggested that off-switching speed correlates well with the amount of achievable switching cycles, reasoning that fast-switching limits the number of photons that can be absorbed and thereby reduces photodestruction [22,32]. However, we estimate that the observed differences in switching speed only contribute to a 22% (rsGreen1) and 42% (rsGreenF) increase in the stabilization of the A state, whereas eight- and three-fold stabilizations are effectively observed (Appendix C, $k_{\mathrm{OA}}$, Table A4). This indicates the presence of an additional mechanism for stabilization of the fast-switching species A by the Enhancer.

## 3. Discussion

We set out to determine whether the direct fusion of the Enhancer nanobody to the rsGreen series of photochromic fluorescent proteins resulted in similar advantages as described previously for non-photochromic FPs [23,29]. While in vitro fusion indeed resulted in analogous spectroscopic improvements in terms of molecular brightness and pH stability, a decrease in fluorescence signal was observed in living cells. We attribute this reduction in apparent brightness in situ to altered expression, degradation and/or maturation of the protein in the complex cellular environment, as is well known to occur for fluorescent proteins [22].

Interestingly, nanobody fusion also affected the photochromism of the labels, increasing the off-switching rate of both rsGreen1 and rsGreenF. This potentially makes the rsGreen-Enhancer fusions superior candidates for RESOLFT sub-diffraction imaging, since fast off-switching allows for short pixel dwell times, which in turn lead to increased temporal resolution [32,33]. To test this hypothesis, future experiments should probe the performance of Enhancer-bound rsGreens in RESOLFT-type imaging compared to the unbound rsGreens and other existing labels. Additionally, the fact that binding of a protein partner changes the switching kinetics opens up the possibility for Enhancer binding to serve as a contrast mechanism for advanced imaging techniques which can discern fluorescent labels with different photochromic behavior, such as lock-in detection [34,35,36], multitau (mt)-pcSOFI [37] or τ-RESOLFT [38]. A highly interesting prospect in this direction is that modulation of photochromism through binding with interaction partners could revolutionize biosensor design by providing a (ratiometric and super-resolution) readout mechanism, which is easy to multiplex with other fluorescence measurements due to limited usage of the visible spectrum [39,40]. A similar modulation of photochromic behavior has recently been published in a biosensor design that enables the visualization of protein kinase A activity with sub-diffraction resolution [28].

Fusions of Enhancer to rsGreen1 and rsGreenF also showed a remarkable increase in their resistance to photoswitching fatigue, as seen by the increase in the amount of achievable switching cycles. This effect was considerably stronger than expected simply based on the increase in photoswitching rate, indicating an intrinsic increase in stability. The reduced photoswitching fatigue could directly benefit any imaging technique that makes use of these labels, since it means more images can be obtained before photodestruction makes the sample unusable. For most imaging techniques this results in the ability to measure the same sample for a longer time. This is illustrated by GMars-Q and rsEGFP2, two reversibly switchable fluorescent proteins that allow prolonged RESOLFT-type imaging owing to their increased fatigue resistance [32,33]. In SOFI, additionally, more recorded images result in an increased signal to noise ratio [41], making it possible to achieve higher resolutions [42,43].

Finally, close examination of the photoswitching behavior suggested that the fluorescent rsGreens and nanobody fusions exist as two spectroscopically different and interconverting molecular species, each with different photochromic behavior. Furthermore, the Enhancer nanobody appears to influence both species, changing their spectroscopic properties while preferentially stabilizing the fast-switching state. This might in turn explain the diminished conversion to the slower-switching species upon repeated photoswitching. The generation of a slower-switching species during repetitive photoswitching also provided an explanation for the increasing baseline fluorescence, which is sometimes observed when using RSFPs, without the need for an additional non-photoswitchable species [33].

Since the forms discovered in this work readily interconvert during the course of a (photoswitching) experiment, caution is warranted when interpreting the findings of such experiments. For instance, it is possible that species B is responsible for most of the signal in RESOLFT and pcSOFI measurements while the in vitro characterization is preformed on a mixture consisting mainly of species A. This information should be taken into account when correlating the performance of the measurement to the molecular parameters, which raises the question whether the presence of several species with distinct switching behavior is a general feature of (green) RSFPs. Likewise, if the technique needs to be tuned to a particular kinetic behavior, this tuning can become progressively worse over the duration of the experiment. Moreover, some applications might favor the use of one state over the other, depending if fast and complete switching (e.g., RESOLFT) or slower switching with a significant on-fraction (e.g., pcSOFI) is preferred. Luckily, since binding of the Enhancer exerts an effect on the occupancy of both species it seems reasonable to assume that one type of photoswitching behavior can be selected for by changing the environment of the chromophore. This could be achieved by either the development of novel RSFPs, through interaction of an existing RSFP with a (suitably modified) version of the Enhancer, or by a combination of both approaches. Shifting the occupancy of chromophore states with different photochromism through binding by a modified Enhancer further intensifies the idea of it serving as a contrast mechanism, as was mentioned above. In any case, a detailed characterization of both species at the molecular level should provide useful information to guide future developments [15,16,17,18,19,20,21].

**Figure 6:**
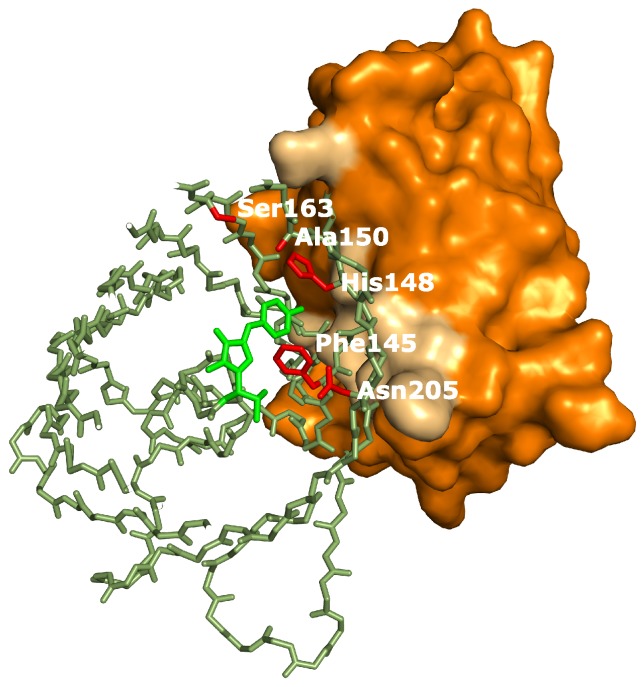
rsGreen0.7 (Protein Data Bank (PDB) accession code: 4xow) aligned to replace green fluorescent protein (GFP) in the structure of Enhancer:GFP (PDB accession code: 3k1k). Colors used: Enhancer (orange, interacting surface in light-brown), rsGreen0.7 (dark-green, chromophore in light-green, residues important for photoswitching in red). In the structure of rsGreen1 and rsGreenF all residues shown are identical to rsGreen0.7, except for the following mutations: L44M, K101E, F145L only in rsGreenF, N149D only in rsGreen1, K162R and H169L.

In summary, five distinct effects of binding by the Enhancer where detected: a drop in the $pK_{a}$, a pH independent increase of extinction coefficient, an increase in off-switching speed (Appendix A), a slower thermal recovery from the off-state and an increased stability of the fast-switching species (Appendix C). Therefore we hypothesize that additional structural changes are induced by Enhancer binding. Crystal structure determination of on- and off-states of rsGreen0.7 [22], rsEGFP2 and rsFolders [19], all closely related to the RSFPs used in this work, identified several key amino acids affecting the photoswitching behavior. Interestingly, the positions occupied by these amino acids correspond very well with the binding site of the Enhancer nanobody [23], as is apparent in Figure 6. The identity of residue 145, for example, was found to influence the photochromism by affecting the chromophore flexibility and is directly involved in Enhancer binding [22]. Additionally, Enhancer binding induces slight structural changes within loop region 142 to 148, shifting His148 into close proximity with Arg168. This might facilitate proton abstraction from the chromophore, therefore favoring the anionic state of the chromophore which in turn affects the photoswitching behavior [44]. Lastly, the Enhancer nanobody is involved in non-polar interactions with a hydrophobic patch on GFP that neighbors residue 205, which was found to be involved in a backbone shift facilitating *cis-trans* isomerization. The bound Enhancer possibly affects this structural change, potentially promoting this backbone shift and thus the photochromic behavior.

The close relationship between the amino acids important for photoswitching and nanobody binding might be a good starting point for understanding the change in photoswitching behavior seen upon enhancer binding. However, to acquire deeper insight into the mechanism by which the Enhancer influences photochromism, the effect of the nanobody on other GFP-based RSFPs should be studied in detail. Additionally, acquiring crystal structures for Enhancer-bound rsGreen1 and rsGreenF will provide a clearer view on the structural rearrangements induced by nanobody binding.

Interestingly, several other proteins exist that selectively bind to fluorescent proteins which could potentially also serve to influence the photochromic behavior of these labels [24,28,45]. For instance, the same work that reported the Enhancer also disclosed a second GFP-binding nanobody that influences spectroscopic properties [23]. This “Minimizer” nanobody has an opposite effect, stabilizing the neutral chromophore state instead of the anionic form, thereby reducing the brightness upon 488 nm excitation. As both the neutral and anionic chromophore states play an important part in the photoswitching mechanism, it is reasonable to expect that binding of the Minimizer nanobody will also induce a change in the photoswitching kinetics of rsGreens.

Taken together, our work demonstrates a novel way to affect the photochromism properties of RSFPs through binding of selected protein motifs, which is a promising prospect regarding a wide variety of advanced fluorescence imaging applications and future protein design.

## 4. Materials and Methods

### 4.1. Cloning, Expression and Purification

The amino acid sequence for Enhancer nanobody was adapted from the Protein Data Bank (Accession number 3K1K, chain A. Deposition authors: Kirchhofer et al. [23]), translated into a Homo Sapiens codon optimized DNA sequence using the Integrated DNA Technologies (IDT, Haasrode, Belgium) codon optimization tool (Web-based tool to be found at https://eu.idtdna.com/CodonOpt) and was ordered in the form of a gBlock^®^ Gene Fragment from IDT (for full amino acid sequence, see Supplementary Materials). The genes encoding rsGreen1 and rsGreenF in the expression vector pRSETb were spliced directly to the Enhancer nanobody by polymerase chain reaction (PCR) driven splicing by overlap extension (SOE) [46]. Final PCR-amplified Enhancer-fusion cDNA contained a *BamHI* restriction site at the 5${}^{\prime }$ end and an *EcoRI* restriction site at the 3${}^{\prime }$ end for insertion into pRSETb in frame with a polyhistidine sequence and into a vector for mammalian expression (pcDNA3). All the used PCR primers are listed in Table 2.

All plasmid transformations were performed by a sonoporation protocol. Chemocompetent *E. coli* JM109(DE3) and DH5α cells (Promega, Leiden, The Netherlands) were incubated with 5 μL plasmid DNA for 5–15 min on ice, followed by sonoporation in a Branson 2210 ultrasonic cleaner for 15 s and incubation with SOC medium for 20 min at 37 ${}^{\circ }$C and were finally plated on bacterial growth plates supplemented with a suitable selection antibiotic.

Expression was conducted by *E. coli* JM109(DE3) strains through leak expression of the T7 promoter of the inserted genes within the pRSETb expression vector. A fresh colony was inoculated in 200–300 mL LB (Luria Bertani) medium supplemented with 100 μg/mL ampicillin and incubated for three successive days (60–72 h) at 23 ${}^{\circ }$C in a thoroughly shaking incubator. Bacterial suspensions were centrifuged for 15 min at 4 ${}^{\circ }$C at 5000 rpm (Sorvall Evolution RC with SLA-1500 rotor). *E. coli* cells were lysed using a french pressure cell press, followed by centrifugation of the cell-debris for 10 min at 4 ${}^{\circ }$C at 8000 rpm with a Biofuge primoR (DJB Labcare ltd, Buckinghamshire, UK). The supernatant containing the crude protein extract was incubated on ice with 2 to 3 mL Ni${}^{2+}$-nitrilotriacetic acid (Ni-NTA) agarose resin (Qiagen, Antwerp, Belgium) and was allowed to bind the protein for 30 min while regularly shaking. This suspension was transferred to a 2 mL polystyrene column (Thermo Fisher Scientific, Merelbeke, Belgium) and washed with excess TN buffer (100 mM Tris-HCl, 300 mM NaCl, pH 7.4). Proteins were eluted with TN buffer supplemented with 100 mM imidazole. Buffer exchange to fresh TN buffer was performed with a PD-10 desalting column (GE Healthcare, London, UK) and protein solutions were stably stored at 4 ${}^{\circ }$C for the duration of the experiments.

### 4.2. In-Vitro Characterization: Spectra, Molecular Brightness and pH Dependence

Absorption spectra of purified FPs were recorded using a Shimadzu UV-1650PC spectrophotometer (Shimadzu GmbH, Duisburg, Germany), while excitation and emission spectra were acquired with an Edinburgh FLS980 spectrometer (Edinburgh Instruments Ltd., Livingston, UK) with a slit widening set at 2 nm. Spectra were measured in TN buffer (pH 7.4).

Extinction coefficients ($\varepsilon_{7.4}$) were determined according to Ward’s method [47], using the literature value of EGFP as a reference [30]. Extinction coefficients ($\varepsilon_{7.4}$) were extrapolated to the extinction coefficient of the deprotonated state ($\varepsilon_{\mathrm{on}}$) by using the Henderson-Hasselbalch equation. Quantum yield (QY) was determined relative to EGFP (=0.60 [30]). Molecular brightness was defined as the product of $\varepsilon_{7.4}$ and $\Phi_{\mathrm{FL}}$, scaled to 100 for rsGreen1.

pH sensitivity measurements were conducted in PBS buffer supplemented with 50 mM citric acid (monoḣydrate), 50 mM KH${}_{2}$PO${}_{4}$ and 50 mM glycine, adjusted to the desired pH. The $pK_{a}$ was determined as the inflection point of the sigmoidal curve that was fitted to the absorption maxima of the anionic form at pH values ranging from 3 to 11.

### 4.3. In Situ Characterization: Brightness and Photoswitching

Transformed *E. coli* JM109(DE3) cells expressing the different FPs were plated on equally sized surfaces with 15 μL bacterial suspension and incubated for 72 h at 20 ${}^{\circ }$C or 24 h at 37 ${}^{\circ }$C. Growth plates were illuminated with a MAX-302 xenon lamp (Asahi Spectra, Tokyo, Japan) and fluorescence was detected with a Cascade 512B CCD camera (Photometrics, Tuscon, AZ, USA). Excitation wavelengths for off-switching and on-switching were selected by a 480/40 nm and 400/30 nm band pass filter, respectively. Emission wavelengths were selected using a 530/40 nm bandpass filter.

Photoswitching of *E. coli* colonies was conducted by submitting growth plates to one full switching cycle comprising 20 periods on-switching, 40 periods off-switching and again 20 periods on-switching, each period lasting 30 s and the emission recorded between periods with a camera exposure time of 0.5 s.

Acquired images were analyzed using Igor Pro 7 (Wavemetrics, Portland, OR, USA). A threshold was set to select all fluorescent colonies and an average intensity trace over the duration of the experiment was calculated and normalized to the point of complete on-switching. The maximum brightness of each separate colony was determined and used to calculate an average brightness value. The maximum fluorescence of every colony was chosen over the average fluorescence to minimize thresholding artifacts.

HeLa cells were cultured in DMEM supplemented with 10% (*v/v*) FBS, 1% (*v/v*) glutaMAX${}^{\mathrm{TM}}$ and 0.1% (*v/v*) gentamicin (all Gibco, Merelbeke, Belgium). Prior to transfection, 250,000–500,000 cells were seeded in 35 mm glass bottom dishes (MatTek, Ashland, MA, USA). The transfection was performed using the FuGene 6 (Promega, Leiden, The Netherlands) transfection protocol. Briefly, 3 μL FuGene 6 was thoroughly mixed with 1 μg DNA (pcDNA3) to a total volume of 100 μL and incubated at room temperature for 20 min. The DNA mixture was then added in dropwise fashion to the medium on top of the cells. After incubation for 20–24 h, cells were washed twice with 2 mL PBS (pH 7.4) and finally supplied with 2 mL HBSS (Gibco, Merelbeke, Belgium) prior to imaging.

High-power photoswitching in HeLa cells was measured on a setup comprising an Olympus IX 71 inverted microscope (Olympus, Berchem, Belgium) coupled to a Spectra X Light Engine (Lumencor, Beaverton, OR, USA), equipped with a 10× objective (UplanSApo, Olympus, Berchem, Belgium), a dichroic turret wheel mounting a ZT488RDC (Chroma, Olching, Germany) dichroic filter with a 535/30 bandpass emission filter. Fluorescence images were recorded with an iXon Ultra 897 EMCCD camera (Andor, Belfast, UK) operating at −70 ${}^{\circ }$C and were processed and analyzed using Igor Pro 7. Images were acquired over 0.05 s during illumination with 1% cyan excitation light. Proteins were submitted to multiple switching cycles comprising 25 periods on-switching with violet light, followed by 25 periods of off-switching with cyan light, each period lasting 100 ms.

Optimal power settings that could be applied for all FPs in a consequent fashion were assessed by recording multiple switching cycles while varying the cyan light source output from 5% to 30%. Twenty percent was determined as the optimal power setting. The decrease in on-state fluorescence was assessed over the course of 500 switching cycles using 7% violet and 20% cyan light. The average on-state fluorescence of the first switching cycle was used as a measure for brightness in HeLa cells as reported in Table 1.

For the measurement of light-independent recovery from the off-state, proteins were submitted to a slightly different illumination scheme consisting of two cycles comprising 5 periods of on- and off switching during 500 ms, followed by acquisition every 30 s during the course of 10 min.

Global fitting was done according to Appendix B using a build-in function of Igor Pro 7.

## Figures and Tables

**Table 1:** Spectroscopic properties of enhanced green fluorescent protein (EGFP), rsGreens and Enhancer constructs. From top to bottom: peak excitation wavelength ($\lambda_{\mathrm{ex}}$), peak emission wavelength ($\lambda_{\mathrm{em}}$), $pK_{a}$, quantum yield of fluorescence ($\Phi_{\mathrm{FL}}$), apparent extinction coefficient ($\varepsilon_{7.4}$) (at $\lambda_{\mathrm{ex}}$, pH 7.4) and the same value extrapolated to the deprotonated form ($\varepsilon_{\mathrm{on}}$), molecular brightness ($\varepsilon_{7.4}\cdot \Phi_{\mathrm{FL}}$), *E. coli* brightness of colonies grown at 37 ${}^{\circ }$C, HeLa brightness. All brightness values are rescaled to 100 for rsGreen1. Values from literature are indicated by the appropriate citation. ND: not determined.

Property	EGFP	rsGreen1	rsGreen1 Enhancer	rsGreenF	rsGreenF Enhancer
$\lambda_{\mathrm{ex}}$ (nm)	489 [30]	487	486	488	485
$\lambda_{\mathrm{em}}$ (nm)	509 [30]	508	505	510	506
$pK_{a}$	5.9	6.2	4.4	6.8	5.6
$\Phi_{\mathrm{FL}}$	0.60 [30]	0.46	0.47	0.42	0.37
$\varepsilon_{7.4}$ ($10^{3}$ L/mol·cm)	52	65	78	52	71
$\varepsilon_{\mathrm{on}}$ ($10^{3}$ L/mol·cm)	53	69	78	65	72
mol. brightness	104 [30]	100	123	73	88
*E. coli* brightness	ND	100	57	82	50
HeLa brightness	ND	100	69	80	51

**Table 2:** List of primer sequences used for amplification and sequencing.

Primer Name	Primer Sequence (5${}^{\prime }$–3${}^{\prime }$)
rsGreen *BamHI* FWD	AA **GGATCC** GATGGTGAGCAAG
rsGreen *PstI* REV	GCAC **CTGCAG** CTTGTACAGCTCGTCCATGC
Enhancer *PstI* FWD	GCTGTACAAG **CTGCAG** GTGCAG
Enhancer *EcoRI* REV∗	AAGCTTC **GAATTC** TTAGGAAACGGT

Bases indicated in bold represent the corresponding restriction site. * denotes that the 3${}^{\prime }$ end codes for a stop codon.

## Supplementary Materials

Supplementary Materials can be found at www.mdpi.com/1422-0067/18/9/2015/s1.

## Abbreviations

(E)GFP	(enhanced) green fluorescent protein
FP	fluorescent protein
(f)PALM	(fluorescence) photoactivated localization microscopy
GFP	green fluorescent protein
IC	Internal Conversion
mt	multi-tau
NL-SIM	non-linear structured illumination microscopy
pcSOFI	photochromic stochastic optical fluctuation imaging
PDB	Protein Data Bank
RESOLFT	reversible saturable optical linear fluorescence transitions
rs	reversibly switchable
sf	superfolder
wt	wild type

## Appendix A. Influence of Molecular Brightness on Photochromism

To determine if the change in the switching kinetics upon Enhancer binding is a direct and logical consequence of the change in the spectroscopic parameters, a model for the kinetics of switching needs to be considered. In this contribution we refer to a simplified model of an excitation cycle given in Equation (A1).

(A1) $$M\overset{\mathrm{excitation}}{⟶}M^{**}\overset{\mathrm{IC}(\mathrm{ps})}{⟶}M^{*}\overset{\mathrm{relaxation}(\mathrm{ns})}{⟶}M$$

In this model the excitation is followed by a fast (picoseconds) relaxation to the vibrational minimum of the lowest excited state through the process of Internal Conversion (IC), from which the molecule can emit light or can non-radiatively return to the ground state in a matter of nanoseconds. In this section we will consider two extreme cases for the mechanism of photoswitching: the photochemical reaction leading to off-switching starts from $M^{*}$, as was often implicitly assumed in the past [15], or switching can only initiate from $M^{**}$, according to recent postulations [20].

When off-switching starts from $M^{*}$, we expect a dependence on the total time spent in the excited state. This means we need to take into account the extinction coefficient (telling us how many times the molecule enters the excitated state) as well as the excited state lifetime of the fluorescent label. Even though the lifetime was not explicitly determined we can use the quantum yield of fluorescence as a proxy, in effect assuming that the changes between the different fluorophores can be accounted for as changes in the rate of non-radiative decay.

When off-switching starts from $M^{**}$, we expect no real dependence on the time spent in the excited state, and thus on the quantum yield of fluorescence. In this case the only thing we need to correct for is a change in extinction coefficient, in effect correcting for the amount of times the molecule enters the excited state.

Unfortunately this model is limited by the fact that the B-species is mostly created in-situ (see Section 2.3 and Appendix B). As such the in vitro measurements of extinction coefficients and quantum yields will reflect mainly on the behavior of the A-species, limiting our corrections to this species. We further neglect the fact that the measured τ values are dictated not only by the rate of off-switching, but also by the rate of on-switching. This can be safely done since the equilibrium on-fraction for the A-species is very low.

(A2) $$\tau_{A,\mathrm{bound},M^{**}}=\tau_{A,\mathrm{unbound}}\frac{\epsilon_{\mathrm{unbound}}}{\epsilon_{\mathrm{bound}}}$$

(A3) $$\tau_{A,\mathrm{bound},M^{*}}=\tau_{A,\mathrm{unbound}}\frac{\epsilon_{\mathrm{unbound}}}{\epsilon_{\mathrm{bound}}}\frac{\Phi_{\mathrm{unbound}}}{\Phi_{\mathrm{bound}}}$$

where $\tau_{\mathrm{bound},M^{**}}$ and $\tau_{\mathrm{bound},M^{*}}$ are the predicted switching lifetimes of the A species (starting from $M^{**}$ and $M^{*}$, respectively), and τ, ϵ and Φ, are the switching lifetime, extinction coefficient and quantum yield for the bound and unbound form, respectively. When we substitute in the measured values from the in vitro characterization (Table 1) and the photoswitching experiment in HeLa cells (Table A2) we obtain the values in Table A1. These lead us to conclude that roughly half of the change in photoswitching kinetics is due to a change in the spectroscopic parameters, meaning some off the change upon binding is due to a second (direct) effect.

**Table A1:** Effect of molecular brightness on switching for the A-species.

Switching lifetime	rsGreen1	rsGreenF
$\tau_{\mathrm{unbound}}$	0.30 cycles	0.25 cycles
$\tau_{\mathrm{bound}}$	0.19 cycles	0.12 cycles
$\tau_{\mathrm{bound},M^{**}}$	0.25 cycles	0.18 cycles
$\tau_{\mathrm{bound},M^{*}}$	0.24 cycles	0.21 cycles

## Appendix B. Two-Species Photoswitching Model

We proposed a model consisting of two distinct and interconvertible fluorescent species, A and B, with time-varying concentrations to explain the experimental photoswitching data. We assumed that the interconversion between A and B is sufficiently slow compared to the photochromism, thus the populations of A and B can be considered to be constant during each single on/off switching cycle. We introduce two variables denoting time: $t_{i}$ denotes the *i*-th on/off photoswitching cycle, and $t_{i}^{\mu }$ denotes image acquisition μ within cycle *i*.

### Appendix B.1. Per-Cycle Model of Switching

For data suitably corrected for background fluorescence, the fluorescence of a single decay occurring at cycle *t* can then be modeled by the following equation

(A4) $$\begin{array}{cc} F(t_{i}^{\mu }) & =A\left(t_{i}\right)\left(1-F_{A}\right)e^{\left(-t_{i}^{\mu }/\tau_{A}\right)} \end{array}$$

(A5) $$\begin{array}{c} +B\left(t_{i}\right)\left(1-F_{B}\right)e^{\left(-t_{i}^{\mu }/\tau_{B}\right)} \end{array}$$

(A6) $$\begin{array}{c} +A\left(t_{i}\right)F_{A}+B\left(t_{i}\right)F_{B} \end{array}$$

where A(t) and B(t) are the signal emitted by species *A* and *B* in the first recorded frame of the off-switching in cycle *i*; $F_{A}$ and $F_{B}$ are the fraction of fluorescence of species A and B that would remain once the on- and off-switching reach steady-state; and $\tau_{A}$ and $\tau_{B}$ are the time constants associated with the disappearance of species A and B, respectively.

This equation explains the experimental data well using a global fitting procedure as seen in Figure 5. It should be stressed that the photoswitching parameters $F_{A}$, $F_{B}$, $\tau_{A}$ and $\tau_{B}$ are kept constant over all of the switching cycles while performing the global fitting procedure. From these fits, we obtain profiles of the underlying concentrations A(t) and B(t), which serve as input for the between cycle model as well as the parameters in Table A2. As these numbers show, the main effect of binding of the Enhancer is a faster off-switching for both A- and B-species.

**Table A2:** Off-switching rate constants (τ) and residual fluorescence (*F*) of the two species.

Photoswitching Parameter	rsGreen1	rsGreen1 Enhancer	rsGreenF	rsGreenF Enhancer
$\tau_{A}$ (frames)	0.3	0.19	0.25	0.12
$\tau_{B}$ (frames)	1.1	0.35	0.72	0.33
$F_{A}$ (%)	0	0	0	0
$F_{B}$ (%)	12	9	10	9

### Appendix B.2. Between Cycle Model of Switching

To explain the observed changes in the populations of A- and B-species the following mechanism is proposed:

(A7) $$\begin{array}{c} \mathrm{bleached}\overset{k_{\mathrm{BA}}}{⟵}A\overset{k_{-1}}{\underset{k_{1}}{⇌}}B\overset{k_{\mathrm{BB}}}{⟶}\mathrm{bleached} \end{array}$$

which can be expressed as the following set of equations

(A8) $$\begin{array}{cc} A^{\prime }(t) & =k_{-1}\chi B\left(t\right)-\left(k_{1}+k_{\mathrm{BA}}\right)A\left(t\right) \end{array}$$

(A9) $$\begin{array}{cc} B^{\prime }(t) & =\frac{k_{1}}{\chi }A\left(t\right)-\left(k_{-1}+k_{\mathrm{BB}}\right)B\left(t\right) \end{array}$$

(A10) $$\begin{array}{cc} A(0) & =A_{0} \end{array}$$

(A11) $$\begin{array}{cc} B(0) & =B_{0} \end{array}$$

where $A_{0}$ and $B_{0}$ are the amount of signal coming from the *A* and *B* species at the start of the experiment, and χ is the ratio of detected signal per molecule of species *A* and *B*.

Unfortunately, in this system several sets of rate constants will lead to the same observed curves. This can be resolved by introducing new aggregate variables that will be unambiguously extractable from the experimental data.

(A12) $$k_{1}^{*}=\frac{k_{1}}{\chi },\;k_{\mathrm{OA}}=k_{1}+k_{\mathrm{BA}},\;k_{-1}^{*}=\chi k_{-1},\;k_{\mathrm{OB}}=k_{-1}+k_{\mathrm{BB}}$$

This system can now be solved easily using e.g., computer algebra software, and when simplified leads to following solutions

(A13) $$\begin{array}{cc} A(t) & =\exp\left(-k_{1,\mathrm{global}}t\right)\frac{A_{0}\left(k_{1,\mathrm{global}}-k_{\mathrm{OB}}\right)-B_{0}k_{-1}^{*}}{k_{1,\mathrm{global}}-k_{2,\mathrm{global}}}\\ & -\exp\left(-k_{2,\mathrm{global}}t\right)\frac{A_{0}\left(k_{2,\mathrm{global}}-k_{\mathrm{OB}}\right)-B_{0}k_{-1}^{*}}{k_{1,\mathrm{global}}-k_{2,\mathrm{global}}} \end{array}$$

(A14) $$\begin{array}{cc} B(t) & =\exp\left(-k_{1,\mathrm{global}}t\right)\frac{B_{0}\left(k_{1,\mathrm{global}}-k_{\mathrm{OA}}\right)-A_{0}k_{1}^{*}}{k_{1,\mathrm{global}}-k_{2,\mathrm{global}}}\\ & -\exp\left(-k_{2,\mathrm{global}}t\right)\frac{B_{0}\left(k_{2,\mathrm{global}}-k_{\mathrm{OA}}\right)-A_{0}k_{1}^{*}}{k_{1,\mathrm{global}}-k_{2,\mathrm{global}}} \end{array}$$

where the following substitutions where used to simplify the equations

(A15) $$\begin{array}{cc} k_{1,\mathrm{global}} & =\frac{k_{\mathrm{OA}}+k_{\mathrm{OB}}}{2}+k_{\mathrm{aggregate}} \end{array}$$

(A16) $$\begin{array}{cc} k_{2,\mathrm{global}} & =\frac{k_{\mathrm{OA}}+k_{\mathrm{OB}}}{2}-k_{\mathrm{aggregate}} \end{array}$$

(A17) $$\begin{array}{cc} k_{\mathrm{aggregate}} & =\sqrt{{\left(\frac{k_{\mathrm{OA}}+k_{\mathrm{OB}}}{2}\right)}^{2}-k_{\mathrm{OA}}k_{\mathrm{OB}}+k_{1}^{*}k_{-1}^{*}} \end{array}$$

From the global fit of curve $A\left(t\right)$ and $B\left(t\right)$ shown in Figure 5 it can be seen that the model explains the data reasonably well leading to the rate constants in Table A3.

**Table A3:** Aggregate rate constants for the two species in the proposed model.

Rate constant	rsGreen1	rsGreen1 Enhancer	rsGreenF	rsGreenF Enhancer
$k_{1}^{*}$ $\left(10^{-3}\;{\mathrm{cycle}}^{-1}\right)$	2.17	0.41	2.69	0.82
$k_{\mathrm{OA}}$ $\left(10^{-3}\;{\mathrm{cycle}}^{-1}\right)$	5.73	0.76	5.93	1.84
$k_{-1}^{*}$ $\left(10^{-3}\;{\mathrm{cycle}}^{-1}\right)$	1.40	0.21	2.13	2.24
$k_{\mathrm{OB}}$ $\left(10^{-3}\;{\mathrm{cycle}}^{-1}\right)$	1.51	0.47	1.45	1.55

From this table we can see that most of the rates of transition between the different states are reduced upon binding the Enhancer, a topic that is further discussed in Appendix C. Care should be taken when comparing values of $k_{1}^{*}$ and $k_{-1}^{*}$ between species, since a change to χ will also change these parameters.

## Appendix C. The Effect of Off-Switching Rate on the Amount of Achievable Switching Cycles

Consider the rate of depletion of the A species ($k_{\mathrm{OA}}$), which is a measure for the amount of achievable switching cycles before loss of suitable contrast between on- and off-state. To detect if a change in $k_{\mathrm{OA}}$ between the Enhancer-bound and unbound form, as seen in Appendix B.2, is directly caused by binding or is an indirect consequence of changes in extinction coefficient, photoswitching speed and quantum yield, we applied Equation (A18). We are limiting ourselves to the A-species because the extinction coefficient and quantum yield for the B-species are unknown. Furthermore, the fraction of residual fluorescence is negligible for species A and the switching is fast enough to be considered complete.

(A18) $$k_{{\mathrm{OA}}^{*},\mathrm{bound}}=k_{\mathrm{OA},\mathrm{unbound}}\frac{\Phi_{\mathrm{bound}}}{\Phi_{\mathrm{unbound}}}\frac{\epsilon_{\mathrm{bound}}}{\epsilon_{\mathrm{unbound}}}\frac{\tau_{A,\mathrm{bound}}}{\tau_{A,\mathrm{unbound}}}$$

where $k_{{\mathrm{OA}}^{*},\mathrm{bound}}$ is the predicted rate of depletion ($k_{\mathrm{OA}}$) of the bound form, and Φ, ϵ and τ are the quantum yield, extinction coefficient and lifetime in their bound and unbound form, respectively. In effect this correction normalizes for the amount of time spent in the excited state. Solving this equation for given parameters gives us the results found in Table A4.

**Table A4:** The effect of molecular brightness and photoswitching on model parameters A-species.

Rate constant	rsGreen1	rsGreenF
$k_{\mathrm{OA},\mathrm{unbound}}$	5.73	5.93
$k_{\mathrm{OA},\mathrm{bound}}$	0.76	1.84
$k_{{\mathrm{OA}}^{*},\mathrm{bound}}$	4.45	3.42

It is clear that the correction we expect for the difference in spectroscopic and photokinetic parameters is insufficient to explain the difference that is seen upon Enhancer binding.
