# Supplementary material for: Reduced Fluorescent Protein Switching Fatigue by Binding-Induced Emissive State Stabilization
Source: Int J Mol Sci. 2017 Sep 20;18(9):2015. doi: 10.3390/ijms18092015 (PMC5618663; doi:10.3390/ijms18092015)
Supplement: Supplementary file 1 [file ijms-18-02015-s001.pdf]

SUPPLEMENTARY MATERIALS TO

Reduced Fluorescent Protein Switching Fatigue by Binding-Induced Emissive State Stabilization

Thijs Roebroek, Sam Duwé, Wim Vandenberg, Peter Dedecker

SUPPORTING METHODS

Amino acid sequences. Fusions of rsGreen1 and rsGreenF to Enhancer nanobody. Green letters indicate fluorescent protein residues, orange letters indicate Enhancer residues[1], leucine indicated in black is an extra residue incorporated for cloning purposes.

rsGreen1-Enhancer:

MVSKGEELFTGVVPILVELDGDVNGHKFSVRGEGEGDATNGKLTMKFICTTGKLPVPWPTLVTTLTYGVLCFARYP
DHMKQHDFFKSAMPEGYVQERTISFEDDGYYKTRAEVKFEGDTLVNRIELKGIDFKEDGNILGHKLEYNFNSHDAYI
TADKQKNGIRSNFKIRLNVEDGSVQLADHYQQNTPIGDGPVLLPDNHYLSTQNKLSKDPNEKRDHMVLLEFVTASG
ITLGMDELYKLQVQLVESGGALVQPGGSLRLSCAASGFPVNRYSMRWYRQAPGKEREWVAGMSSAGDRSSYEDS
VKGRFTISRDDARNTVYLQMNSLKPEDTAVYYCNVNVGFEYWGQGTQVTVS

rsGreenF-Enhancer:

MVSKGEELFTGVVPILVELDGDVNGHKFSVRGEGEGDATNGKLTMKFICTTGKLPVPWPTLVTTLTYGVLCFARYP
DHMKQHDFFKSAMPEGYVQERTISFEDDGYYKTRAEVKFEGDTLVNRIELKGIDFKEDGNILGHKLEYNLNSHNAYI
TADKQKNGIRSNFKIRLNVEDGSVQLADHYQQNTPIGDGPVLLPDNHYLSTQNKLSKDPNEKRDHMVLLEFVTASG
ITLGMDELYKLQVQLVESGGALVQPGGSLRLSCAASGFPVNRYSMRWYRQAPGKEREWVAGMSSAGDRSSYEDS
VKGRFTISRDDARNTVYLQMNSLKPEDTAVYYCNVNVGFEYWGQGTQVTVS

SUPPLEMENTARY TABLES AND FIGURES

Supplementary Table 1. Brightness of rsGreen1, rsGreenF and Enhancer fusions when expressed in *E.coli* grown at 37°C and 20°C. From left to right: mean brightness, standard deviation, standard error on the mean, Welch's t-test: t-value, degrees of freedom, p-value.

					**Welch's t-test**		
	**mean brightness**	**std.dev.**	**SEM**	**N**	**t**	**df**	**p**
37°C							
**rsGreen1**	9927,19	1914,90	228,88	70	17,4712	91	<0,0001
**rsGreen1-Enhancer**	5615,80	949,91	92,26	106			
**rsGreenF**	8151,56	865,73	166,61	27	15,7288	53	<0,0001
**rsGreenF-Enhancer**	4955,88	1046,50	116,28	81			
20°C							
**rsGreen1**	10663,00	1912,09	291,59	43	1,4848	75	0,1418
**rsGreen1-Enhancer**	9981,51	2213,66	354,47	39			
**rsGreenF**	8897,09	2136,20	280,50	58	0,4383	58	0,6628
**rsGreenF-Enhancer**	8669,64	2507,73	436,54	33			

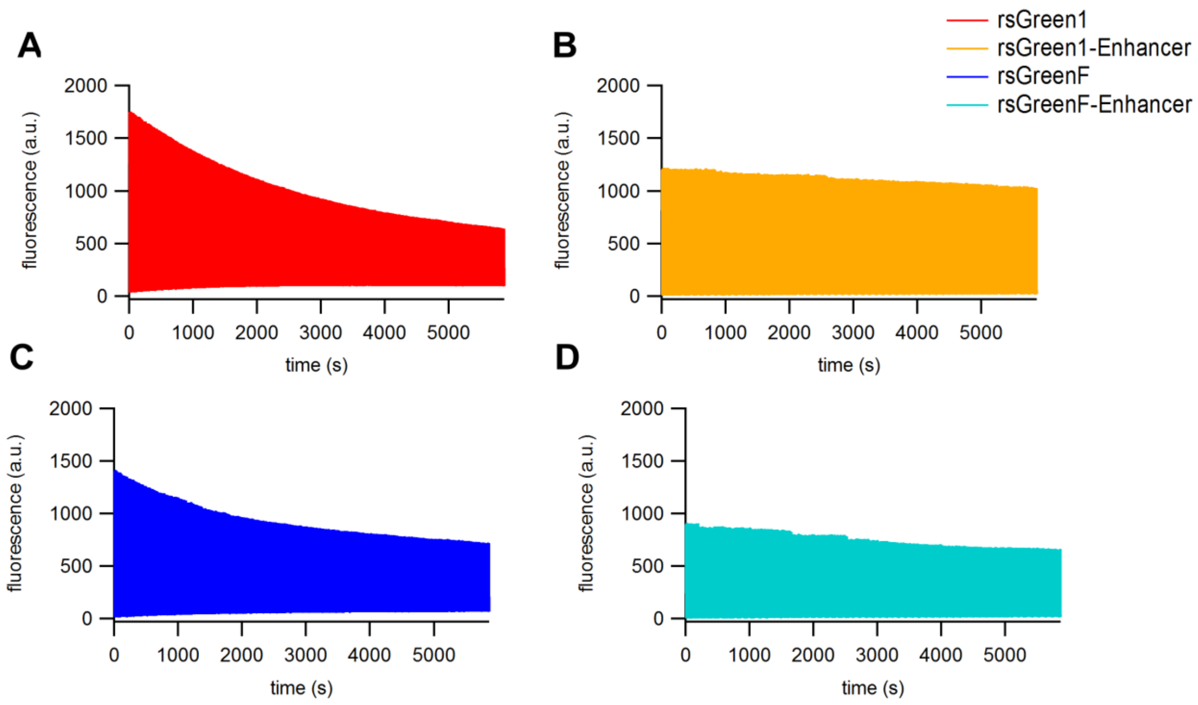

Supplementary Figure 1. Representative fluorescence photoswitching traces of rsGreen1 (A), rsGreen1-Enhancer (B), rsGreenF (C), rsGreenF-Enhancer (D) expressed in HeLa cells and submitted to 500 consecutive switching cycles.

SUPPORTING REFERENCES

1. Kirchhofer A, Helma J, Schmidthals K, et al. Modulation of protein properties in living cells using nanobodies. *Nat Struct \& Mol Biol*. 2010;17(1):133-U162. doi:10.1038/nsmb.1727.
